# Differential gut microbiome in spondyloarthritis patients associated to *Blastocystis* colonization

**DOI:** 10.1038/s41598-023-39055-z

**Published:** 2023-08-18

**Authors:** Carlos Nieto-Clavijo, Liliana Morales, Ricaurte Alejandro Marquez-Ortiz, Consuelo Romero-Sánchez, Alejandro Ramos-Casallas, Javier Escobar-Perez, Wilson Bautista-Molano, Juan Manuel Bello-Gualtero, Jacqueline Chaparro-Olaya

**Affiliations:** 1 Laboratorio de Parasitología Molecular, Vicerrectoría de Investigaciones, Universidad El Bosque, Edificio O. Segundo Piso, Avenida Carrera 9 #131A-02, Bogotá, Colombia; 2 Bacterial Molecular Genetics Laboratory, Universidad El Bosque, Bogotá, Colombia; 3 Cellular and Molecular Immunology Group (InmuBo), Universidad El Bosque, Bogotá, Colombia; 4 Clinical Immunology Group, School of Medicine, Universidad Militar Nueva Granada-Hospital Militar Central, Bogotá, Colombia; 5 Rheumatology and Immunology Department, Hospital Militar Central, Bogotá, Colombia

**Keywords:** Microbiome, Spondyloarthritis, Parasitology

## Abstract

The role of *Blastocystis* in intestinal health is an open controversy, and little is known about the potential effect of this microorganism in autoinflammatory diseases such as spondyloarthritis (SpA). Here, we analyzed the gut microbiome of 36 SpA patients and 13 control individuals and demonstrated that the richness, diversity, and taxonomic composition between these two groups are different. We also showed that colonization by *Blastocystis* in control individuals increases the richness and diversity of the intestinal microbiome, whereas in SpA patients, it does not seem to have any impact. This may reflect a potential role of *Blastocystis* in sculpting the gut microbiome architecture in control individuals, whereas in subjects with SpA, the modulation of the microbiome may be governed by disease-dependent factors that cannot be overcome by *Blastocystis*. Regarding taxonomic characterization, SpA patients colonized by *Blastocystis* showed significant increases in the phylum *Pseudomonadota*, class *Gammaproteobacteria*, family *Succinivibrionaceae*, and genus *Succinivibrio*. Simultaneously, there were significant increases in the class *Bacilli*, order *Lactobacillales*, families *Lactobacillaceae* and *Clostridiaceae,* and genera *Lactobacillus* and *Clostridium* in non-colonized SpA patients. On the other hand, PICRUSt analysis in *Blastocystis*-positive SpA patients showed elevations in pathways that may enhance antioxidant capacities and alleviate intestinal inflammation, while *Blastocystis*-negative SpA patients showed significant changes in pathways that promote cell division/proliferation and can lead to larger changes in the gut microbiome. Our analyses lead us to believe that these changes in the gut microbiome of SpA patients may trigger protective mechanisms as an initial response to inflammation in an attempt to restore balance in the intestinal environment.

## Introduction

*Blastocystis* has a worldwide distribution and is often the most prevalent intestinal protist in humans. *Blastocystis* occurs with a prevalence of up to 24% in developed countries^1^, although a study in 100 healthy Irish adults found a prevalence of 56%^2^. In Colombia, recent reports show a variable prevalence between 12 and 90%^3–8^.

The role of *Blastocystis* in human health is still controversial. The parasite has been associated with nonspecific gastrointestinal symptoms (such as abdominal pain, constipation, diarrhea, fatigue, flatulence, nausea, vomiting and weight loss), irritable bowel syndrome (IBS), Inflammatory bowel disease (IBD) and skin disorders^9–12^. However, *Blastocystis* has also been found in the intestinal flora of healthy people (without any clinical manifestation), even more frequently than in individuals with gastrointestinal symptoms or IBD^13,14^. In addition, some studies have found that *Blastocystis* does not play a role in the activation of ulcerative colitis or IBS, but conversely, it could have a protective role^15^. On the other hand, there is growing evidence for a connection between *Blastocystis* colonization and an increased richness of the gut microbiome in healthy carriers (compared to healthy noncarriers)^16^, although these modifications do not always behave similarly in patients^17^. Clearly, the debate is still open.

Spondyloarthritis (SpA) is a family of chronic inflammatory joint diseases that includes ankylosing spondylitis (AS), reactive arthritis (ReA), psoriatic arthritis (PsA), SpA associated with inflammatory bowel disease (SpA-IBD), undifferentiated SpA (uSpA) and juvenile SpA (JSpA)^18^. Several studies have shown that the microbial profiles of SpA patients and healthy individuals are different, and there are increasing numbers of reports on the relationship between the gut microbiome and SpA^19–23^. Simultaneously, a relationship between *Blastocystis* and variations in the intestinal microbiome has been proposed, since greater bacterial diversity and richness have been found in individuals carrying the parasite. These findings have suggested *Blastocystis* as a member of the healthy intestinal microbiota^2,14,24,25^.

To participate in the debate, the aim of this study was to evaluate the potential effects of colonization by *Blastocystis* on the gut microbiome of SpA patients and healthy individuals.

## Methods

### Study population

A cross-sectional study was carried out in which nonprobabilistic convenience sampling was used. Stool samples from 15 healthy individuals (control individuals) and 40 patients fulfilling the ASAS criteria for SpA were collected^26^. Ten control subjects and 20 SpA patients’ samples were from a previous study^27^. Experienced rheumatologists enrolled the remaining 20 SpA patients from March 2020 to November 2020 at the Hospital Militar Central and Clínicos IPS (Bogota, Colombia), and five additional control individuals with ages and socioeconomic conditions similar to those of the SpA patients were included. The functional index and disease activity for each patient were assessed using BASFI (Ankylosing Spondylitis Functional Index), BASDAI (Bath Ankylosing Spondylitis Disease Activity Index), and ASDAS-CRP (Ankylosing Spondylitis Disease Activity score using C-reactive protein)^28,29^.

Pregnancy, infection during the last month, autoimmune diseases, neoplasia, immunodeficiency, chronic pancreatitis, liver disease, diabetes, age under 18 and over 65 years and antibiotic treatment, systemic steroid treatment or antiparasitic treatment during the last 3 months were the exclusion criteria. All SpA patients presenting gastrointestinal symptoms underwent a thorough evaluation to rule out IBD. Symptoms indicative of inflammatory diarrhea, including the presence of mucus, rectal bleeding, tenesmus, defecatory urgency, and abdominal pain with pseudo-obstructive characteristics, were examined. Additionally, potential signs of IBD were excluded by evaluating perianal abnormalities and conducting both endoscopic and histological examinations to detect macroscopic findings such as ulcerations, penetrations, erosions, or strictures, as well as microscopic alterations in crypt architecture or crypt abscesses. For participants who underwent a colonoscopy, the stool sample was taken before bowel preparation. None of the control individuals presented gastrointestinal symptoms at the time of sample collection.

Prior to inclusion in the study, written informed consent for study participation was obtained from all participants (SpA patients and control individuals). Corporate Research Ethics Committee of Hospital Militar Central (Bogotá, Colombia) approved this research (Minute 09 of June 1, 2018 and Minute 03 of February 15, 2019), which was conducted in accordance with the principles outlined in the Declaration of Helsinki and guidelines established by the Ministerio de Salud y Protección Social de Colombia for research involving human subjects (Resolution number 8430 of 1993).

### Stool processing

A single fresh stool sample was collected from each participant and transported to “Universidad El Bosque” while maintaining the cold chain at 4 °C. Each sample was divided into four aliquots before processing. The first aliquot was immediately processed for intestinal parasites detection by microscopy. The second aliquot was stored at 4 °C for fecal calprotectine (FCP) measure. The third aliquot was concentrated and stored at − 20 °C for parasite detection by PCR, while the fourth one was stored at − 80 °C for bacterial microbiome analysis. DNA extraction from aliquots stored at − 20 °C and − 80 °C were performed within two weeks after sample collection.

### FCP and CRP measures

For each participant, a quantitative FCP measurement was performed by ELISA, using the KAPEPKT849 kit (DIAsource ImmunoAssays S.A., Louvain-la-Neuve, Belgium). A blood sample was used to measure CRP by chemiluminescence immunoassay (Immulite 1000. Siemens Healthineers, Erlangen, Germany). The established normal cut-off point for FCP and CRP was ≤ 120 ng/ml and < 3 mg/L, respectively.

### Microscopic detection of intestinal parasites

A fresh stool sample from each patient was analyzed by microscopic examination of a wet mount (0.9% saline solution and 4% lugol), concentrated sample (Mini-Parasep SF system. DiaSys. Wokingham, UK) and Kato-Katz (Sterlitech. Washington, USA). The attending physician made the treatment decision for participants who tested positive for intestinal parasites.

### Molecular detection of *Blastocystis*, *Giardia intestinalis*, *Entamoeba histolytica* and *Cryptosporidium*

Fresh feces were concentrated and stored at − 20 °C for a maximum of two weeks before processing. DNA from 200 μL of each sample was extracted using the QIAamp DNA Stool Mini Kit (Qiagen. Germantown, USA). Successful DNA extraction was confirmed by qPCR for *Enterobacteriaceae* 16S rRNA gene as described previously^30^.

*Blastocystis* and *Giardia intestinalis* colonization was also evaluated by qPCR of the 18S rRNA gene using primers, probes and conditions reported before^31,32^ (Table S1).

A semi-nested PCR on the 18S rRNA gene was done to identify *Entamoeba histolytica* using primers described previously^32,33^ (Table S1) according to the method reported before^27^.

*Cryptosporidium* was also detected by a semi nested PCR of the 18S rRNA gene. Primers Crip_F and Crip_R were used in the first PCR reaction^34^ and primers ChvF18S^35^ and Crip_R^34^ in the second one (Table S1). The PCR mix contained 2 mM MgCl_2_, 0.2 mM dNTPs, 0.2 μM of each primer, 1μL template DNA, and GoTaq® G2 Flexi DNA Polymerase (Promega. Madison, USA). The amplification program for both reactions was 95 °C for 3 min, 35 cycles at 95 °C for 30 s, 60 °C for 30 s and 72 °C for 30 s and a final extension at 72 °C for 7 min.

Primers and TaqMan probes were synthesized by Macrogen (Seoul, Korea). PCR reactions were performed on a T100 PCR thermal cycler (BioRad. Hercules, USA) and qPCR assays were run on a CFX96 Touch real-time PCR system (BioRad. Hercules. USA).

### Statistical analysis

Descriptive statistics (mean, median, standard deviation and interquartile ranges) were used to characterize the study population. Assumption of normality was evaluated using the Shapiro–Wilk test. Group comparisons were performed using a mean-difference Z test to continuous variables conforming to a normal distribution; if not, a Wilcoxon rank-sum test was applied. For comparing proportions or categorical variables, Z-proportion or chi-squared tests were performed. A *p-*value < 0.05 was considered statistically significant. Statistical analyses were performed using STATA V.16.1. (StataCorp LLC, College Station, USA).

### Microbiome analysis

Fresh feces were stored at − 80 °C for a maximum of two weeks before processing. DNA from 200 μg of each sample was extracted using the QIAamp Power Fecal Pro DNA kit (Qiagen. Germantown, USA), following the manufacturer's recommendations. The extracted DNA was used to amplify the V3-V4 16S rRNA regions by PCR using the Bakt_341F and Bakt_805R primers^36^ (Table S1) and Herculase II Fusion DNA Polymerase (Agilent). Nextera DNA libraries (Nextera XT Index Kit V2. Agilent) were prepared using the PCR products, and amplicon sequencing was performed (by Macrogen) on a MiSeq Illumina platform following a standard protocol for paired-end reads of 300 nucleotides. The resulting sequencing data were analyzed using the QIIME2 pipeline^37^. DADA2 was used to discard the first 10 nucleotides from the 5' end and the last 20 nucleotides from the 3' end. Trimmed reads were merged and filtered to remove low-quality data and finally clustered as amplicon sequence variants (ASVs).

ASVs were classified and assigned to taxonomic levels using the Greengenes database (release v. 13.8)^38^. The Chao1 index (species richness), Shannon index (diversity of species) and Faith's PD index (phylogenetic diversity) were calculated to explore the alpha diversity. Comparisons of these three indices between SpA patients and control individuals were performed with an ordinary unpaired t-test. When *Blastocystis* colonization was taken into account, four subgroups were defined: *Blastocystis*-positive control individuals (*C* +), non-colonized controls (*C−*), *Blastocystis*-positive SpA patients (*P* +) and non-colonized SpA patients (*P−*). ANOVA and Tukey’s multiple comparisons test were used to compare alpha diversity among these subgroups. Statistical analyses were carried out on GraphPad Prism V.9 (GraphPad Software, La Jolla, California, USA).

Beta diversity was assessed by principal coordinate analysis (PCoA) based on Bray‒Curtis distances. Statistical validation through PERMANOVA (using 1000 permutations) was determined with QIIME2 and used to estimate *p*-values for differences among groups and subgroups in PCoA analyses.

For taxonomic characterization, significant differences in taxa abundance between groups were established in STAMP v2.0.9^39^ using White’s nonparametric test. The relative abundance of a given taxon was assumed to be significantly different if the *p*-value was < 0.05 and the absolute value of the difference of the means exceeded 0.3% (arbitrary value). Benjamini‒Hochberg FDR correction was applied to account for multiple comparisons^40^. A heat map was generated by using Heatmapper (http://www.heatmapper.ca/)^41^.

### PICRUSt analysis

PICRUSt analysis^42^ was conducted to determine the predicted metabolic functions of the microbial communities in SpA patients and control individuals colonized or not by *Blastocystis.* The predicted metagenomes (the contribution of each ASV to the overall gene content of the sample) were categorized by metabolic function in MetaCyc, and significant differences were established with White’s nonparametric t test in STAMP. Benjamini‒Hochberg FDR correction was applied to account for multiple comparisons. Differences between proportion means > 0.03% (arbitrary value) with a *p*-value < 0.05 were assumed to be significant.

### Ethics declarations

This study was conducted in accordance with the principles outlined in the Declaration of Helsinki and guidelines established by the Ministerio de Salud y Protección Social de Colombia for research involving human subjects (Resolution number 8430 of 1993). Prior to inclusion in the study, written informed consent for study participation was obtained from all participants (SpA patients and control individuals). Corporate Research Ethics Committee of Hospital Militar Central (Bogotá, Colombia) approved this research (Minute 09 of June 1, 2018 and Minute 03 of February 15, 2019).

## Results

### Population and intestinal parasite infection

Since the aim of this study was to evaluate the potential effects of *Blastocystis* on the gut microbiome, and functional and compositional changes in the intestinal microbiota have been demonstrated during the course of *G. intestinalis* and *E. histolytica* infections^43^, four patients and two control subjects positive for these parasites were excluded from the analysis. Therefore, the study population was 36 SpA patients and 13 control individuals. Among the SpA patients, 72.2% (n = 26) were AS patients, 19.5% (n = 7) were PsA patients and 8.3% (n = 3) were ReA patients. According to the BASDAI and BASFI indices, 75% (n = 27) and 64% (n = 23) of the SpA patients presented active disease and functional limitation, respectively. The ASDAS-CRP score revealed active disease in 31 SpA patients (86%). The demographic and clinical characteristics of the participants are summarized in Table 1.

**Table 1:** Demographic and clinical characteristics of SpA patients control subjects.

Variable	SpA patients	Control subjects	*p-*value
	n = 36	n = 13	
Sex (n, %)			
Female	14–38.88	6–46.15	0.648^a^
Male	22–61.11	7–53.85	
Age (years), mean ± SD	42.56 ± 11.05	43.92 ± 15.03	0.730^b^
Height (m), mean ± SD	1.67 ± 0.07	1.65 ± 0.11	0.433^b^
Weight (kg), median (IR)	70.50 (65.50–79.50)	70.00 (57.00–80.00)	0.659^c^
BMI (kg/m^2^), mean ± SD	25.89 ± 3.35	25.80 ± 4.88	0.939^b^
Smoker (n, %)			
Yes	2–5.56	1–7.69	0.783^d^
No	34–94.44	12–92.31	
BASDAI, mean ± SD	5.70 ± 2.69		
BASFI, mean ± SD	5.28 ± 2.92		
ASDAS-CRP, mean ± SD	2.71 ± 0.95		
GI symptoms* (n, %)			
Diarrhea (**)	17–47.22		
Blood in stool	6–16.67		
Mucus in stool	8–22.22		
Abdominal pain	23–63.89		
Abdominal distention	20–55.56		
Treatment (n, %)			
Non-biological drugs	14–38.89		
Methotrexate	1–2.78		
Sulfasalazine	3–8.33		
NSAID	10–27.78		
Biological drugs	18–50.00		
Anti-TNF (ADA)	11–30.55		
Anti-TNF (ETA)	5–13.89		
Anti-TNF (GLM-CTZ)	2–5.56		
Anti-IL-17	2–5.56		
No treatment	4–11.11		

(**a**) Z-test for proportions was used to calculate differences of proportions. (**b**) t-test was used to calculate differences of means. (**c**) Wilcoxon rank sum was used to calculate differences of medians (**d**) Chi-Square was used for testing relationships on categorical variables.

*SpA* Spondyloarthritis, *SD* standard deviation, *IR* interquartile range, *BMI* body mass index, *BASDAI* Bath Ankylosing Spondylitis Diseases Activity Index, *BASFI* Bath Ankylosing Spondylitis Functional Index, *ASDAS-CRP* Ankylosing Spondylitis Disease Activity Score using CRP, *GI* gastrointestinal, (*) none of the control individuals presented gastrointestinal symptoms at the time of sample collection, (**) Diarrhea lasting more than 4 weeks, *NSAID* Non-steroidal anti-inflammatory drug, *ADA* Adalimumab, *ETA* Etanercept, *GLM* Golimumab, *CTZ* Certolizumab.

In SpA patients and control subjects, *Endolimax nana* was the most prevalent parasite (80.56% and 92.31%, respectively), followed by *Blastocystis* (63.89% and 61.54%, respectively). Z-test proportion analysis showed no significant differences in the frequency of *E*. *nana* (*p* = 0.353) or *Blastocystis* (*p* = 0.880) between groups. In addition, *Entamoeba coli* (n = 3), *Chilomastix mesnili* (n = 3) and *Entamoeba hartmanni* (n = 1) were also identified in SpA patients. None of the SpA patients or control subjects had helminth or *Cryptosporidium* infection (Table S2). In summary, the study population consisted of 36 SpA patients (23 colonized by *Blastocystis* and 13 *Blastocystis* free) and 13 control individuals (eight colonized by *Blastocystis* and five *Blastocystis* free).

### FCP and CRP measures

Median concentration of FCP in the SpA patient group was higher (54.44 ng/ml. IR: 43.79–188.11) compared to that in the control group (42.70 ng/ml. IR: 40.0–89.4). When considering *Blastocystis* colonization in the analysis, SpA patients without *Blastocystis* had higher FCP levels (168.34 ng/ml. IR: 49.7–260) than those colonized by *Blastocystis* (51.83 ng/ml. IR: 41.75–107.09). The same when analyzing the AS patient group independently, in which the median FCP concentrations were 168.34 ng/ml (IR: 50.12–265.85) and 48.98 ng/ml (IR: 42.08–102.99) for non-colonized and colonized AS-patients, respectively. Although these differences were not statistically significant, the median concentration of FCP in patients (SpA or AS) without *Blastocystis* was found to exceed the established normal threshold of 120 ng/ml, whereas in patients colonized by *Blastocystis* it remained below that value. Finally, 83.4% of the SpA patients and 84.6% of the control individuals exhibited normal CRP values with median concentrations of 0.78 mg/L (IR: 0.3–2.38) and 0.96 mg/L (IR: 0.35–1.21), respectively**.** This difference was not statistically significant.

### Alpha diversity, Beta diversity and disease activity and functional indices (BASDAI and BASFI)

A total of 6397 ASVs were found in the libraries with a mean frequency of 10,843 (ranging from 4776 to 15,698). The rarefaction curve indicated sufficient sampling depth, reaching saturation. To establish alpha diversity, Chao1 (which estimates the number of species—richness—), Shannon (which gives a measure of both richness and evenness—diversity—) and Faith's PD (which correlates with the number of species and corresponds to phylogenetic richness) indices were used.

**Figure 1:**
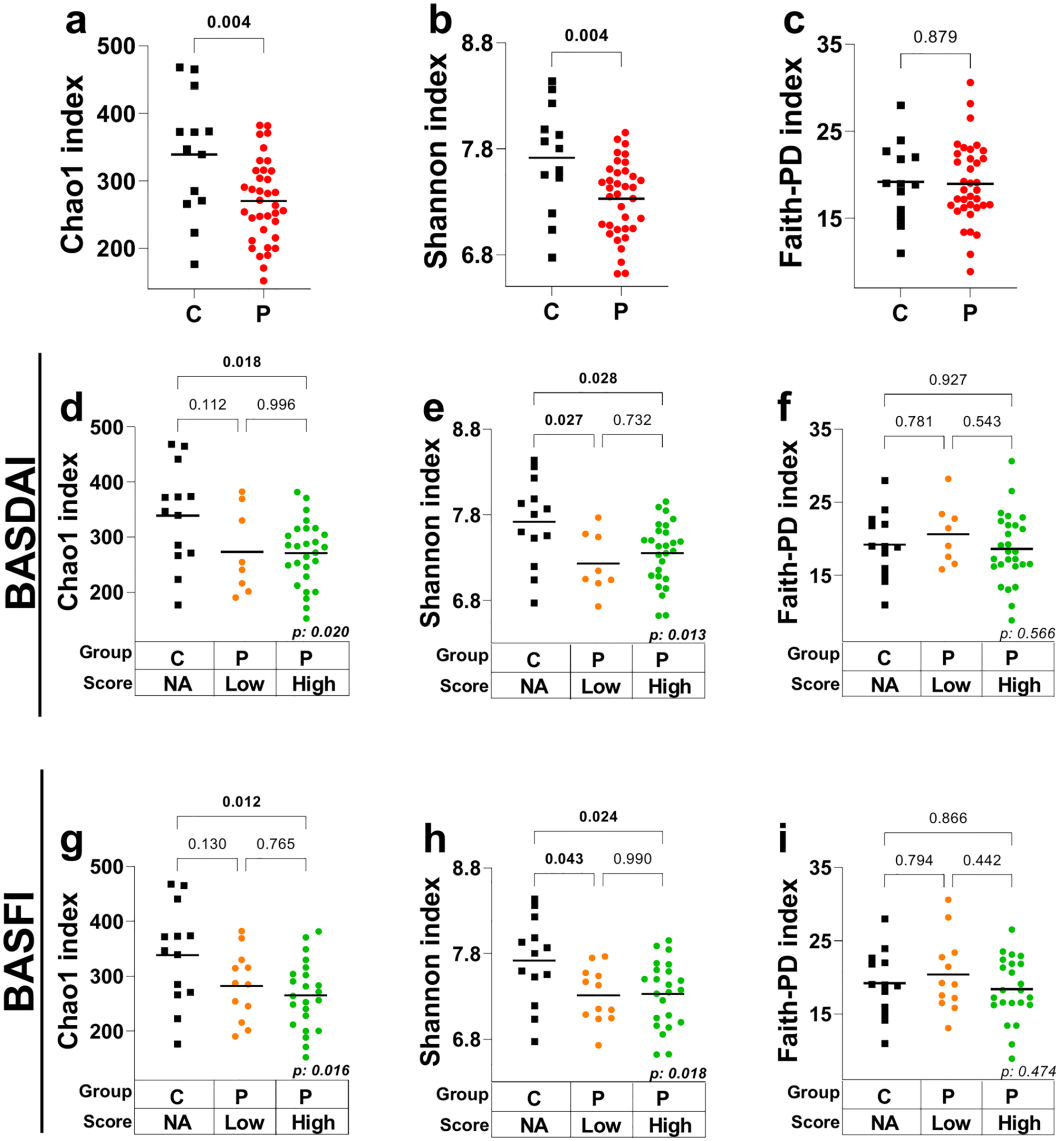
Comparisons of microbiome composition in SpA patients and control subjects. Composition according to BASDAI and BASFI indices. *C* control individuals, *P* SpA patients, *Low* BASDAI or BASFI indices < 4 (less disease activity or functional limitation), *High* BASDAI or BASFI indices ≥ 4 (more disease activity or functional limitation), *NA* not applicable. Significant differences (*p*-value < 0.05) are shown in bold. An unpaired t-test was used to compare groups in panels (**a**–**c**) An ANOVA test (*p*-value in the lower right corner of panels (**d**–**i**) and a Tukey's multiple comparisons test were performed to compare subgroups.

Significantly higher values of richness (Chao1 index. *p* = 0.004) and bacterial diversity (Shannon index. *p* = 0.004) were found in the control group compared to the SpA patient group (Fig. 1a,b; Table S3). In contrast, phylogenetic richness assessed by Faith’s PD index (*p* = 0.879) was similar in both groups (Fig. 1c; Table S3).

Differences between Chao1 and Faith’s PD indices suggest that the gut microbiome in control subjects is enriched with rare species, which have lower weight in Faith’s PD index.

When the microbiome analysis was based on the disease activity and functional indices (BASDAI and BASFI), significant differences in richness (Chao1 index; *p* = 0.018 and *p* = 0.012, respectively, Fig. 1d,g; Table S3) and diversity (Shannon index; *p* = 0.028 and *p* = 0.024, respectively, Fig. 1e,h; Table S3) were observed between control individuals and SpA patients with high disease activity or high functional limitation (BASDAI or BASFI scores ≥ 4.0). Comparisons between control subjects and SpA patients with low disease activity or low functional limitation (BASDAI or BASFI scores < 4.0) revealed significant differences in the Shannon index only (*p* = 0.027 and *p* = 0.043, respectively) (Fig. 1e,h; Table S3).

No significant differences in alpha diversity were observed between SpA patients, regardless of their disease activity or functional limitation scores (Fig. 1d–i; Table S3). Finally, phylogenetic richness (Faith PD index) was similar among all groups (Fig. 1f,i; Table S3).

To test beta diversity, PCoA plots were created using the Bray‒Curtis measure in QIIME2. The pseudo F Permanova test on BASDAI (*p* = 0.004) and BASFI (*p* = 0.011) scores showed significant differences between control individuals, SpA patients with high disease activity or functional limitation, and SpA patients with low disease activity or functional limitation (Fig. S2a,c). Subsequent pairwise analysis between subgroups showed significant differences between control individuals and SpA patients, regardless of their BASDAI or BASFI scores (low or high) (Fig. S2b,d). When comparing between SpA patients with low and high BASDAI scores, they behaved as significant different groups (*p* = 0.032). This was not the case for SpA patients with low and high BASFI scores (*p* = 0.145) (Fig. S2b,d). These findings suggest that the relationship between disease activity and the microbiome differs from the relationship between functional impairment and the microbiome in SpA patients.

### Alpha diversity and colonization by Blastocystis

When *Blastocystis* colonization was taken into account, four subgroups were defined: (*C* +), (*C−*), (*P* +) and (*P-*). In the subsequent analyses, four comparisons were considered: *C* + versus *C−*, *P* + versus *P−*, *C* + versus *P* + and *C−* versus *P−*. The comparisons between *C* + and *P−* or between *C−* and *P* + were not explored, since if significant differences were found between them, it would not be possible to establish whether the findings were related to the disease, the presence of the parasite or both.

**Figure 2:**
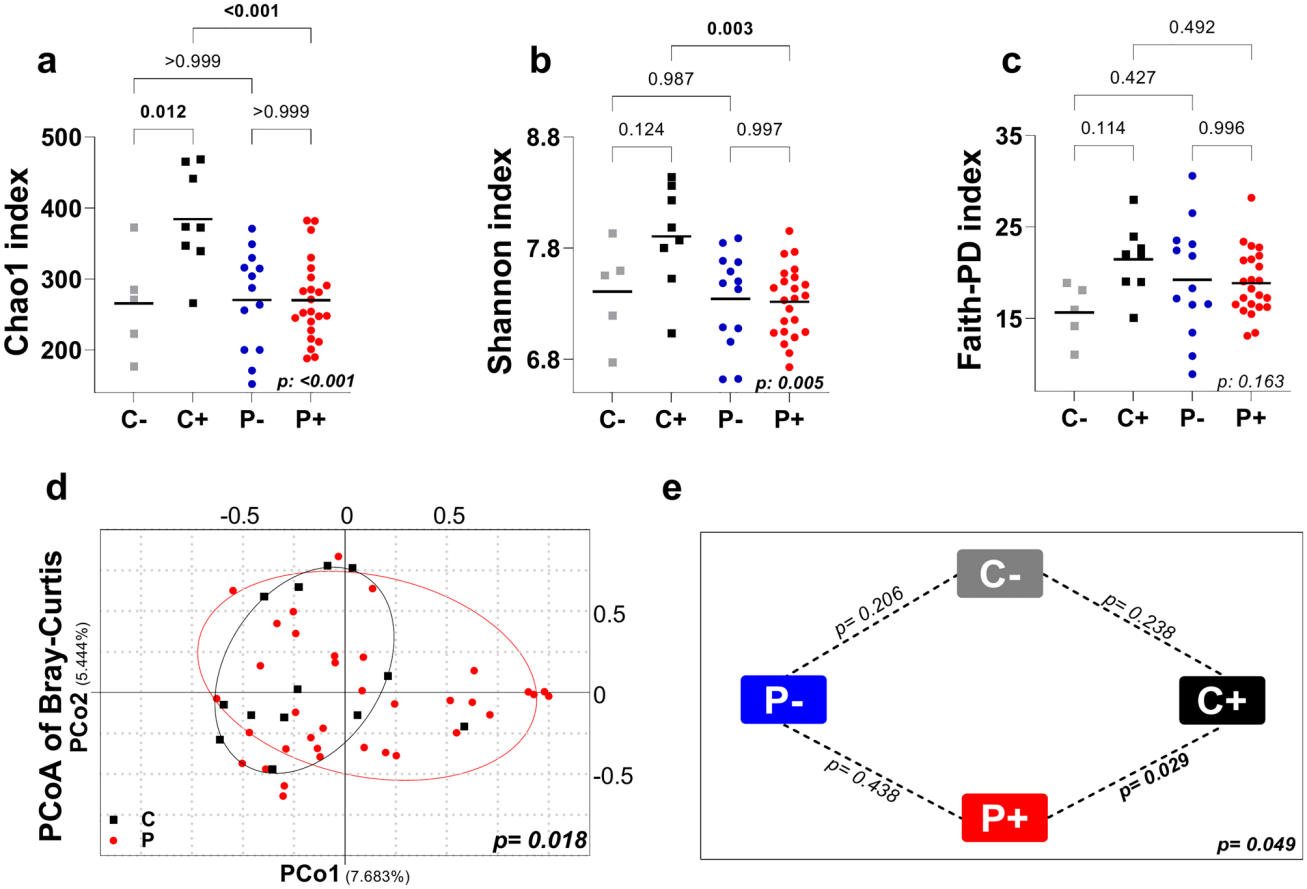
Alpha and beta diversity of gut microbiome in control individuals and SpA patients, colonized and not colonized with *Blastocystis*. (**a**) Chao1. (**b**) Shannon diversity. (**c**) Faith-PD index. (**d**) Bray–Curtis dissimilarity Principal Coordinate Analysis (PCoA) based on bacteria community features between SpA patients and control subjects. (**e**) *p*-values from PCoA between subgroups. *C − *control subjects *Blastocystis* free, *C* + control subjects colonized by *Blastocystis*, *P − *SpA patients *Blastocystis* free, *P* + SpA patients colonized by *Blastocystis*. Significant *p-*values (< 0.05) are shown in bold. A Tukey’s multiple comparisons test was used to compare groups in panels (**a**–**c**). PERMANOVA analysis was used to compare differences in beta diversity between groups in panels (**d**,**e**).

When control individuals were classified according to the presence of *Blastocystis* (*i.e.*, *C* + versus *C*−), colonized individuals showed a significant increase in the Chao1 index (*p* = 0.012), suggesting that the enrichment previously observed in control individuals (compared to SpA patients) might be due to a cross-talking between *Blastocystis* and the local microbiota (Fig. 2a; Table S3). Alpha diversity comparisons among SpA patients showed no significant differences between *P* + and *P−* individuals (Fig. 2a–c; Table S3).

Comparisons among *Blastocystis-*positive subjects (*i.e., C* + versus *P* +) showed a significant increase in the Chao1 (*p* < 0.001) and Shannon indices (*p* = 0.003) (Fig. 2a,b; Table S3) in control individuals. In contrast, there were no significant differences in the Chao1 or Shannon indices among *Blastocystis*-negative subjects (i.e.,* P−* versus *C−*) (Fig. 2a–c; Table S3).

All these results further validate a significant decrease in the richness and diversity of the gut microbiome in SpA patients versus control individuals. Interestingly, our findings suggest that colonization in control individuals, but not in SpA patients, increases bacterial richness and diversity.

### Beta diversity

To test beta diversity, PCoA plots were created using the Bray‒Curtis measure in QIIME2. Individuals were grouped into two significantly different clusters (*p* = 0.018) (Fig. 2d) composed of control individuals or SpA patients. When the four defined subgroups were analyzed (i.e.,* C* +,* C−, P* + and *P−*), the pseudo F Permanova test revealed a significant difference between them (*p* = 0.049). Subsequent paired analysis between subgroups showed a significant difference only between *C* + and *P* + (*p* = 0.029) (Fig. 2e). This suggests that colonization with *Blastocystis* significantly contributes to the observed difference between the control group and the SpA patients.

### Microbiome taxonomic characterization

**Figure 3:**
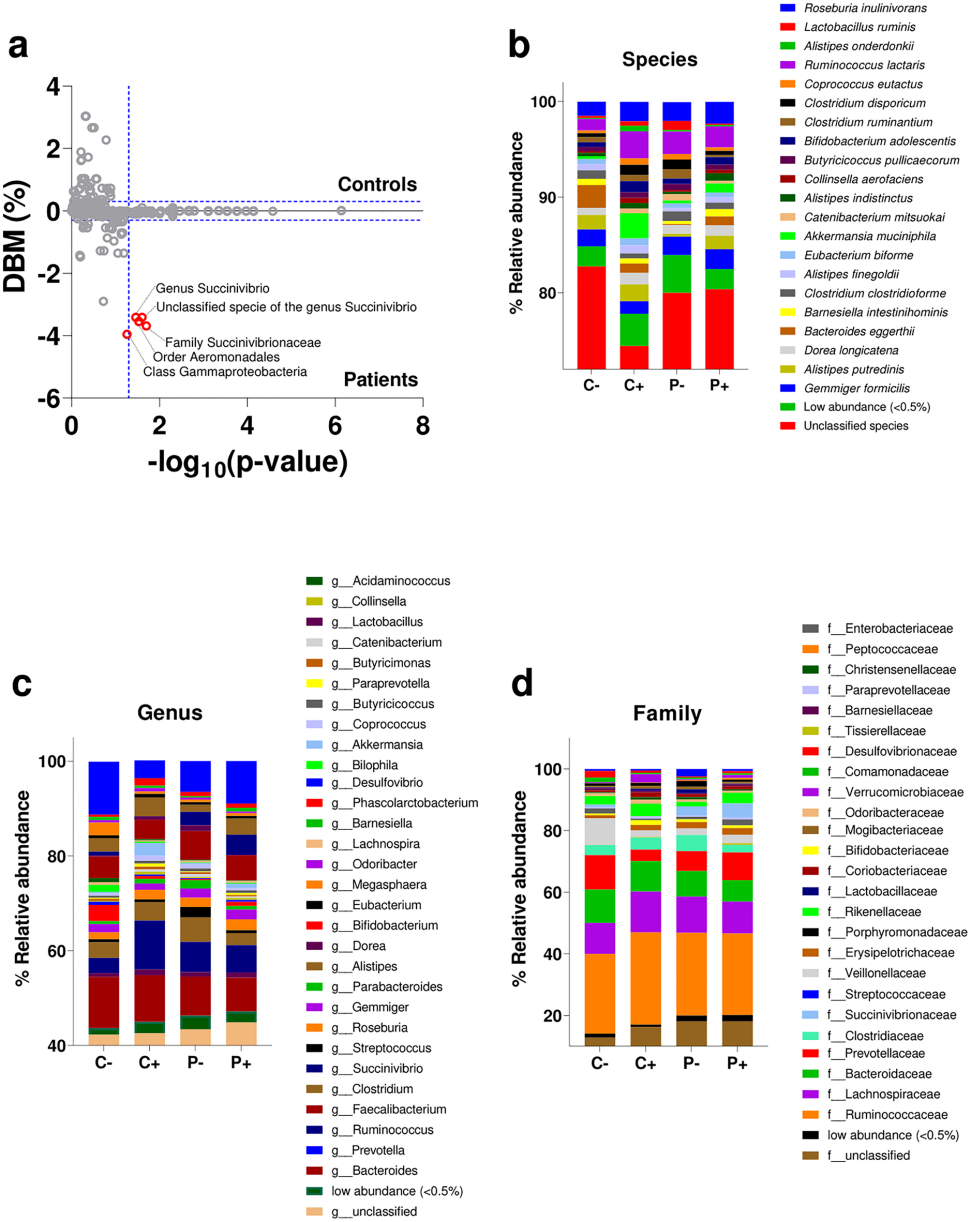
Taxonomic characterization for gut microbiome in SpA patients and control individuals, colonized and not colonized by *Blastocystis*. (**a**) Analysis of differential abundance (using differences between means, DBM) between the control group and the SpA patients. Positive DBM values correspond to overrepresented taxa in control individuals. Negative values correspond to overrepresented taxa in SpA patients. Statistically significant taxa according to White's non-parametric t-test paired comparisons are shown in red circles. DBM arbitrary threshold (± 0.3%) and significant *p*-value threshold (< 0.05) are shown in dashed blue horizontal and vertical lines, respectively. (**b**–**d**) Bars represent average relative frequency at different taxonomic levels. Taxa with relative frequency greater than 0.5% are shown. *C − *control subjects *Blastocystis* free (n = 5), *C* + control subjects colonized by *Blastocystis* (n = 8), *P − *SpA patients *Blastocystis* free (n = 13), *P* + SpA patients colonized by *Blastocystis* (n = 23).

As stated before, the relative abundance of a given taxon was assumed to be significantly different if the *p*-value was < 0.05 (Benjamini‒Hochberg FDR correction applied) and the absolute value of the difference of the means exceeded 0.3% (arbitrary value). Figure 3b–d and S1 show the relative abundance of species (Fig. 3b), genera (Fig. 3c), family (Fig. 3d), order (Fig. S1a), class (Fig. S1b) and phylum (Fig. S1c) for the four defined subgroups (*C−, C* +,* P−* and *P* +). A high percentage of unclassified species (∼80%) (Fig. 3b), genera (∼40%) (Fig. 3c) and families (∼20%) (Fig. 3d) was found. At the order, class, and phylum levels, only ~ 5% of the reads were unidentified.

**Figure 4:**
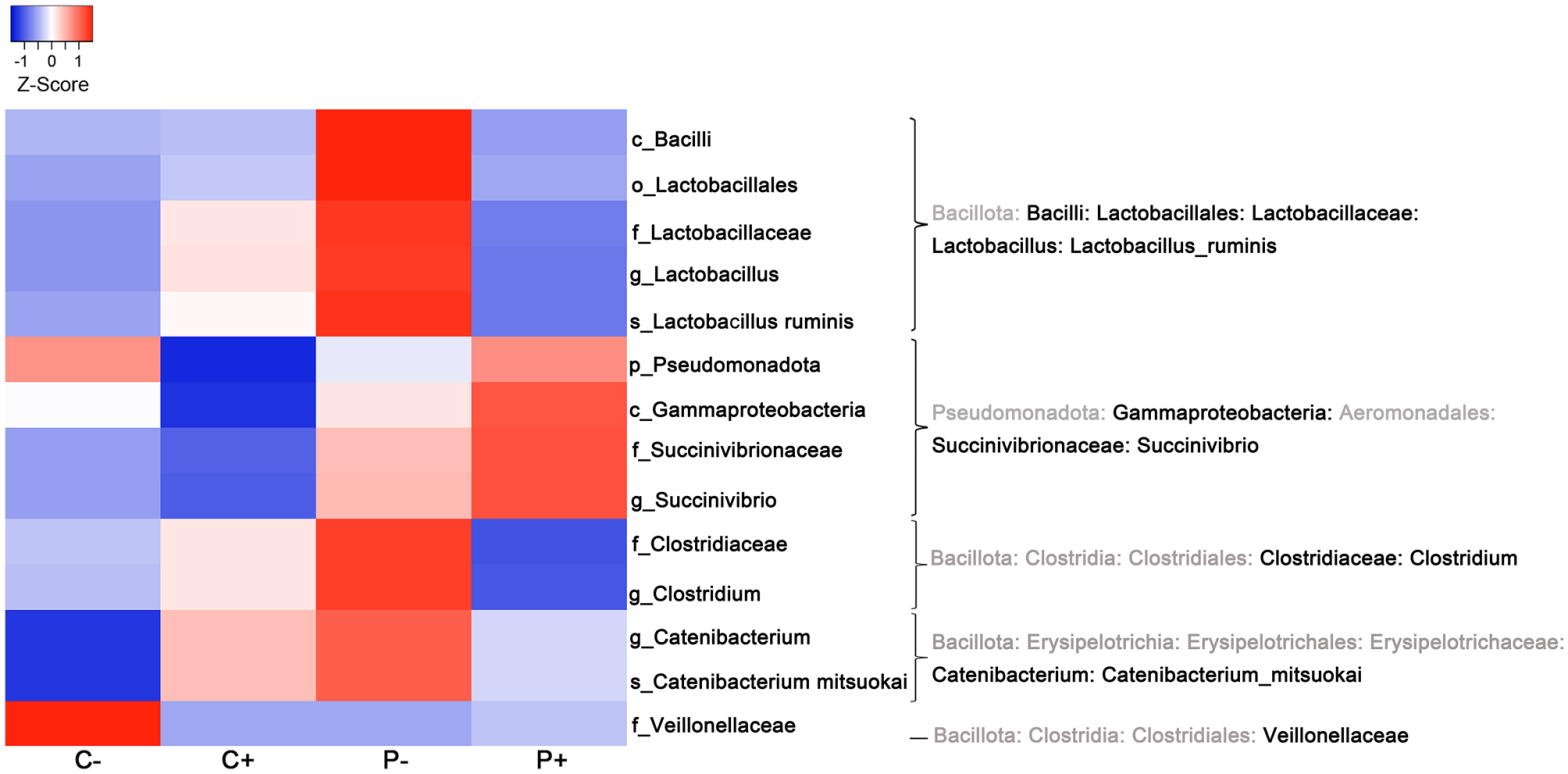
Heat map of the relative abundances of intestinal bacteria among subgroups. In the four subgroups established (*C* +, *C −,* *P* + and *P −*), the analysis of relative abundance revealed significative differences in 14 taxa belonging to five taxonomic hierarchies, shown on the right. The color gradient ranges from the minimum value in blue to the maximum value in red. Rows show amplicon sequence variants (ASV) whose relative abundance was statistically significant. *C − *control subjects *Blastocystis* free, *C* + control subjects colonized by *Blastocystis*, *P − *SpA patients *Blastocystis* free, *P* + SpA patients colonized by *Blastocystis*. The heat map was generated by using Heatmapper^41^.

Relative abundance analysis revealed that in SpA patients, compared with control subjects, there were significant increases in the order *Aeromonadales* (*p* = 0.029), the family *Succinivibrionaceae* (*p* = 0.019), the genus *Succinivibrio* (*p* = 0.034) and an unclassified species from the genus *Succinivibrio* (*p* = 0.025). A higher relative abundance of the class *Gammaproteobacteria* was also observed in SpA patients (5.7) than in the control group (1.73) (Fig. 3a), but it was not statistically significant (*p* = 0.053). Similarly, when *Blastocystis*-positive subjects were analyzed (*P* + versus *C* +), significant increases in the phylum *Pseudomonadota* (*p* = 0.030), the class *Gammaproteobacteria* (*p* = 0.010*)*, the family *Succinivibrionaceae* (*p* = 0.010*)* and the genus *Succinivibrio* (*p* = 0.010*)* (Fig. 4; Table S4) were found in *P* + subjects. No differences in these taxa were found when *Blastocystis*-negative subjects were compared (*P−* versus *C−*) (Table S4), suggesting that the previously observed differences between control subjects and SpA patients (Fig. 3a) may be determined by the colonized individuals. However, the microbiome analysis in *Blastocystis*-negative subjects (*P−* versus *C−*) revealed a significant increase in the class *Bacilli* (*p* = 0.033) and the order *Lactobacillales* (*p* = 0.013) in the *P−* subgroup (Fig. 4; Table S4).

On the other hand, when colonized and non-colonized SpA patients were compared (*P* + versus *P−*), the *P−* individuals showed significant increases in the relative abundances of the class *Bacilli* (*p* = 0.030), the order *Lactobacillales* (*p* = 0.030), the family *Lactobacillaceae* (*p* = 0.040), the genus *Lactobacillus* (*p* = 0.040) and the species *Lactobacillus ruminis* (*p* = 0.040) (Fig. 4; Table S4). Additionally, the family *Clostridiaceae* (*p* = 0.020) and the genus *Clostridium* (*p* = 0.010) were significantly more abundant in *P-* individuals (Fig. 4; Table S4).

Finally, analyzing control individuals (*C* + versus *C−*), a significantly higher proportion of the family *Veillonellaceae* was found in the *C−* subgroup (*p* = 0.050). The genus *Catenibacterium* and its species *mitsuokai* were absent in all individuals of subgroup *C−* (Fig. 4; Table S4), resulting in significant differences with colonized control individuals (*p* = 0.050 and *p* = 0.041, respectively).

### Predicted metagenome functional content

**Figure 5:**
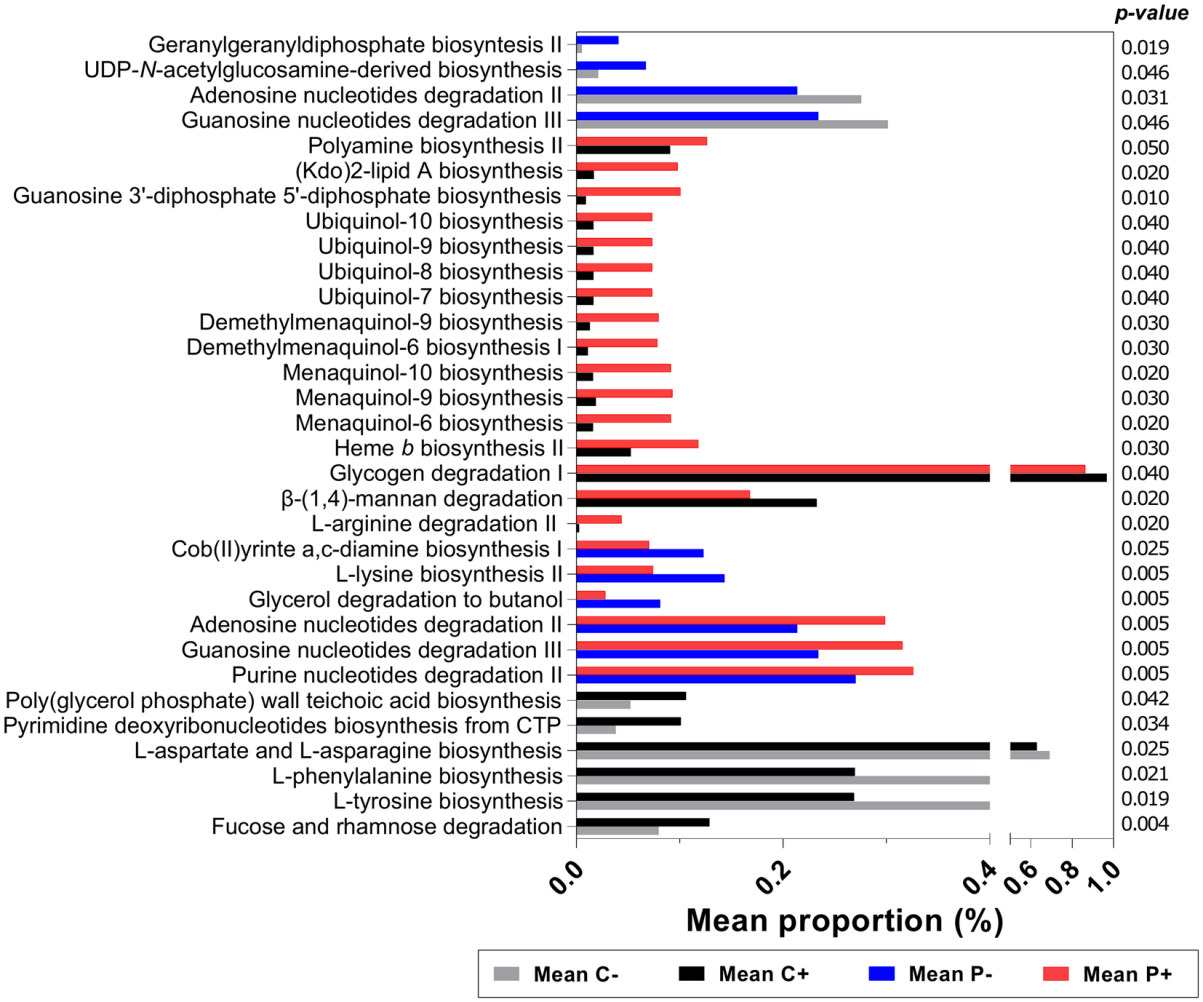
Relative abundances of predicted metabolic pathways for SpA patients and control individuals. The mean relative abundances of the predicted metabolic pathways were compared among the four study subgroups. Metabolic pathways of *C − *subjects are shown in gray, *C* + individuals in black, *P − *subjects in blue and *P* + individuals in red. Statistical significance was obtained by White's nonparametric t-test and Benjamini–Hochberg FDR correction. Comparisons were performed as follows: (*C − *versus *P −*), (*C* + versus *P* +), (*P* + versus *P −*) and (*C* + versus *C −*). Only differences with *p*-value < 0.05 and a DBM thresholds ≥ 0.3% are shown.

Predicted metabolic functions of the microbial communities in subgroups were investigated by using PICRUSt. Metagenomes predicted from the 16S rRNA gene data revealed 387 metabolic pathways, among which 32 showed significant differences (Fig. 5).

There were four significantly different pathways between the *C−* and *P−* subgroups, 16 between *C* + and *P* +, six between *P−* and *P* +, and six between *C−* and *C* +. Among these, eight were degradation pathways, and 22 were biosynthesis pathways. The most common pathways were those for purine degradation and the synthesis of quinones (essential components of respiration), amino acids and teichoic acid (present in the cell wall of gram-positive bacteria).

## Discussion

We analyzed the gut microbiomes of 36 SpA patients and 13 control individuals and demonstrated that the richness, diversity, and taxonomic composition between these two groups were different. Higher gut microbiome richness and diversity were found in control individuals. We also found microbiome differences between individuals colonized by *Blastocystis* and those non-colonized.

### Alpha and beta diversity

Beta diversity analysis established that SpA patients and controls were compositionally distinct groups, with alpha analysis showing significantly lower richness (Chao1 index) and diversity (Shannon index) in the SpA patients. Similar results were found in a meta-analysis of 92 studies in patients with rheumatic diseases, in which a significant decrease in the richness and diversity of the gut microbiome was observed in patients compared to controls^44^.

When comparing *Blastocystis*-positive (*C* +) and *Blastocystis*-negative (*C−*) control individuals, a higher richness of bacterial communities was observed in colonized subjects. Similar results were recently reported in a study in which *alpha* diversity was significantly higher in *Blastocystis*-positive samples. However, in that previous study, there was no information about potential background diseases in the analyzed individuals, and it was not possible to establish associations among health, disease and *Blastocystis*^45^. The same was true for a retrospective study using *Blastocystis*-positive and *Blastocystis*-negative samples in which significantly higher richness and diversity were found in colonized subjects. However, the samples came from individuals with a wide range of pathologies (with and without gastrointestinal symptoms), and it was not possible to establish with certainty a cause/effect relationship between the parasite and the intestinal microbiome^46^.

In contrast, we did not find changes in bacterial richness or diversity when comparing *Blastocystis*-positive (*P* +) and *Blastocystis*-negative (*P−*) SpA patients, which may reflect a different modulation of the gut microbiome in SpA patients, mostly driven by the disease.

### Alpha diversity and disease activity and functional indices

Decreased richness and diversity were observed in SpA patients with high disease activity or high functional limitation compared to control subjects. In contrast, only a significant decrease in diversity was found in SpA patients with low BASDAI or BASFI scores compared to controls. These results may reflect the strong impact of the inflammatory burden on the intestinal microbiota and a kind of progression of dysbiosis. That is, the diversity of the bacterial population decreases at the beginning of the disease, and then, when the disease activity and functional limitation increase, a significant decrease in bacterial richness appears. Indeed, gastrointestinal manifestations are known to be frequent in SpA patients, and inflammation is one of the most important signs. In a previous study on AS patients using a GA-map™ Dysbiosis Test (which grades gut microbiota aberrations on a 1–5 scale), an association was found between gut dysbiosis and higher scores for BASDAI, BASFI and ASDAS-CRP. These associations remained after adjustment for relevant confounders, which suggested that gut dysbiosis may be a biomarker of more severe AS^47^. In agreement with our study, Berland et al. reported a progressive loss of microbial richness associated to increasing disease activity. Upon categorizing SpA patients into two groups based on disease activity levels (low disease activity: BASDAI < 4, and high disease activity: BASDAI ≥ 4), they found significant differences in metagenomic species pangenome (MSP) richness between healthy controls and SpA patients with high BASDAI. Also in line with our findings, no significant differences in MSP richness were observed between healthy controls and SpA patients with low disease activity^48^. In contrast, Sternes et al. defined and analyzed four subgroups of AS patients based on the BASDAI score (0–2.5; 2.5–5.0; 5.0–7.5 and 7.5–10) and observed no significant changes in bacterial richness or diversity among them^49^. We attribute the discordant results to the very dissimilar subgroup size in each study.

### Taxonomic characterization

Our bacterial community analysis revealed that in SpA patients, compared with control subjects, there was a significant increase in the relative abundances of the family *Succinivibrionaceae* and genus *Succinivibrio*. Similarly, comparing *Blastocystis*-positive subjects (*P* + versus *C* +), the *P* + subgroup showed significant increases for the phylum *Pseudomonadota*, the class *Gammaproteobacteria*, the family *Succinivibrionaceae* and the genus *Succinivibrio*. Previous evidence showed that phylum *Pseudomonadota* enrichment is related to dysbiosis and disease status^50^. It has also been suggested that members of *Pseudomonadota* with adherent and invasive properties could lead to the development of IBD^51^. However, studies in mice revealed that although elevations in *Pseudomonadota* correlate with IBD, this effect was mostly due to *Enterobacteria* species^52^. Regarding the *Gammaproteobacteria* class and the *Succinivibrionaceae* family, they have been suggested to play beneficial roles associated with immune patterning and immune recovery, respectively^53,54^. In a study comparing viremic untreated HIV-infected individuals, immunological antiretroviral therapy (ART) responders, ART nonresponders and healthy controls, the *Succinivibrionaceae* family was found to be significantly overrepresented in ART responders. The authors found that members of this family may participate in the active transport and accumulation of molecules implicated in the anti-viral response, inflammation resolution and immune recovery, all of which were found to accumulate inside the bacterial cells of immunological responders^54^. Likewise, members of the genus *Succinivibrio* have been shown to produce succinates that promote tuft cell expansion and resolve intestinal inflammation in mice. The authors demonstrated that the administration of succinate to TNF^∆^ARE/ + mice (mutants with chronic inflammatory arthritis and IBD) and to anti-CD3E-treated mice (another animal model of intestinal inflammation involving infiltrative disease of the small intestine) reduces intestinal inflammation and modulates the immune response in a tuft cell-dependent manner^55^. These findings, however, do not contrast with the known role of succinate in inflammation. Although this metabolite has been shown to accumulate under conditions of dysbiosis, succinate may initially trigger protective mechanisms in response to inflammation and subsequently add to inflammatory processes dysregulating the overall response and contributing to disease. In fact, PICRUSt analysis did not show any imbalance in succinate production, perhaps because the major producers of succinate in the mammalian gut belong to the phylum *Bacteroidota,* and we did not observe any change in these bacteria. PICRUSt also revealed elevations in L-arginine degradation and polyamine biosynthesis pathways in *P* + subjects, both processes involved in low-level succinate production. Also notable was the increased biosynthesis of ubiquinone and vitamin K quinones (menaquinones and methyl-menaquinones), as recent evidence established that vitamin K may improve antioxidant capabilities, alleviate intestinal inflammation, enrich the intestinal bacterial microbiome, recover intestinal integrity and reduce colonic tumor development^56^.

In summary, although it is not possible to establish with certainty whether the increased abundance of *Pseudomonadota*, *Gammaproteobacteria*, *Succinivibrionaceae* and *Succinivibrio* has a protective effect in SpA patients colonized by *Blastocystis*, we hypothesize that these changes could be part of a response initially aimed at restoring balance in the intestinal environment.

On the other hand, the analysis of the gut microbiome in *Blastocystis*-negative subjects (*P−* versus *C−*) revealed significant increases in the class *Bacilli* and the order *Lactobacillales* in the *P−* subgroup. Similarly, when comparing colonized and non-colonized SpA patients (*P* + versus *P−*), *P−* individuals showed significant increases in the same two taxa as well as in the family *Lactobacillaceae*, the genus *Lactobacillus* and the species *Lactobacillus ruminis*. A previous study found that AS patients with increased FCP levels had a higher abundance of the *Bacilli* class and *Lactobacillus* genus^23^. In our study, the median FCP concentration in the *Blastocystis*-free AS patients was higher (168.34 ng/ml. IR: 50.12–265.85) than in the *Blastocystis*-colonized individuals (48.98 ng/ml. IR: 42.08–102.99).

In addition, the mean relative abundances of the *Bacilli* class and *Lactobacillus* genus were higher in the non-colonized subgroup (5.18 and 1.30, respectively) than in the *Blastocystis*-positive subgroup (1.37 and 0.20, respectively). Therefore, our evidence shows that *Blastocystis*-negative AS patients have a higher median concentration of FCP and that, as in the report by Klinberg et al., the *Bacilli* class and the *Lactobacillus* genus are more abundant in them^23^. However, our study was not designed to focus on AS patients or to include the FCP level as a variable. In addition, it is important to consider the different cutoff values used for FCP in each study. In another report, Kim et al. analyzed *Blastocystis*-positive and *Blastocystis*-negative samples and found that *Lactobacilli* and *Bacilli* were very abundant in the *Blastocystis*-negative group. However, again, the results are not directly comparable to ours because Kim et al. performed their analysis based on diarrhea presentation in individuals who visited a hospital but whose medical status was not specified^57^.

Despite the different experimental designs, our results are in agreement with previous reports in which the *Bacilli* class and the genus *Lactobacillus* were found in higher abundance in individuals with a variety of disease states.

*Lactobacillus* and *Bacillus* species have been associated with beneficial effects on intestinal integrity, ecosystem homeostasis and function. Similarly, *Lactobacillus* and *Bacillus* have shown efficacy in the prevention and treatment of intestinal conditions such as diarrhea, IBS, IBD and colorectal cancer^58^. It has also been shown that *L. ruminis* can alleviate dextran sulfate sodium (DSS)-induced colitis in mice by decreasing proinflammatory cytokines, upregulating short-chain fatty acids and improving the *Bacillota* and *Bacteroidota* imbalance caused by DSS^59^.

On the other hand, the family *Clostridiaceae* and the genus *Clostridium* were also significantly more abundant in *P-* individuals. Drawing conclusions about *Clostridiales* is complex. Some of these bacteria may play a role in the induction of regulatory T cells, but their abundance may be the same, higher or lower in individuals suffering from different immune-mediated inflammatory diseases (such as Crohn's disease, ulcerative colitis, multiple sclerosis and rheumatoid arthritis) than in healthy controls^60^.

Although there may be a link between the host immune response and the aforementioned bacteria (*Bacilli, Lactobacillales, Clostridiaceae* and *Clostridium)* in the *P-* subgroup, it is not possible for us to hypothesize about their global effect on the pathogenesis of SpA or their relationship with *Blastocystis* colonization.

Regarding the PICRUSt analyses on *Blastocystis*-free SpA patients, there was a significant decrease in the purine degradation pathways, which suggests their rescue to favor cell division/proliferation. This is consistent with the observed increase in L-lysine biosynthesis, a pathway with a bifurcation leading to peptidoglycan synthesis (the main component of the bacterial cell wall). An increase in the biosynthesis of geranylgeranyl diphosphate, a crucial pathway in the biosynthesis of several terpenes involved in membrane and cell wall synthesis, was also observed. Finally, PICRUSt showed a superabundance of the cob(II)yrinate synthesis pathway, a precursor of cobalamin (vitamin B12). An increase in this pathway and the overgrowth of some bacterial species can disrupt the balance of vitamin K absorption, as bacteria competitively exchange and uptake cobalamin along with glycoprotein intrinsic factors. These events create host-bacteria competition for cobalamin, prevent the absorption of the vitamin into the circulation and lead to larger changes in the gut microbiome^61^. To date, there is not enough evidence to make conclusions about the physiological consequences of *Blastocystis* absence and the overgrowth of some bacterial types in SpA patients.

## Conclusions

Overall, our results validate previous findings related to a significant decrease in gut microbiome richness and diversity in SpA patients versus control individuals. We also showed that *Blastocystis* colonization in control individuals increases gut microbiome richness and diversity, while in patients, it seems to have no impact. This may reflect a different modulation of the gut microbiome in SpA patients, probably driven by their chronic inflammation and altered immune system. The results of the taxonomic characterization and PICRUST analyses suggest that in a disease such as SpA, part of the observed changes in the gut microbiome could be attempts to regain intestinal gut balance. However, these results are not without limitations, one of which is the small number of clinical samples used for analysis.

### Limitations

There are several limitations to the present study, including the aforementioned constraint of a relatively small sample size. First, causal relationships cannot be inferred. We cannot establish whether *Blastocystis* can modulate the bacterial community to exert an effect on SpA or if the bacterial community favors colonization by *Blastocystis.* Second, *Blastocystis* subtypes (STs) could exhibit differential pathogenic potential, and we did not identify *Blastocystis* STs in this work. Associations among *Blastocystis*, disease and dysbiosis have rendered conflicting results, and it has been postulated that *Blastocystis* STs could be responsible for those differences. A recent study showed that in mice infected with *Blastocystis* ST4 or ST7, each ST was associated with opposite changes in the microbiome (ST4 increased beneficial bacteria and ST7 reduced them). They also showed differential production of SCFAs and different colitis outcomes. However, as the authors stated, this is the first report in a murine model, and this finding should be questioned in humans^62^. Third, ~ 90% of the enrolled SpA patients were receiving drug therapy, which could be a factor in gut microbiome remodeling. However, we believe microbiome comparisons among SpA patients are plausible because the treatment distribution was similar in the *P* + and *P−* subgroups. The frequency of biological and non-biological treatment in *Blastocystis*-free patients was 50% and 50%, while in colonized patients, it was 59% and 41%, respectively.

### Supplementary Information

Supplementary Information.

## Data Availability

The datasets generated for this study can be found in NCBI Bioproject PRJNA947188. Other data underlying this article cannot be shared publicly due to the privacy of individuals that participated in the study. The data will be shared on reasonable request, and may be requested via email to romeromaria@unbosque.edu.co.
