# Supplementary material for: Differential gut microbiome in spondyloarthritis patients associated to Blastocystis colonization
Source: Sci Rep. 2023 Aug 18;13:13480. doi: 10.1038/s41598-023-39055-z (PMC10439117; doi:10.1038/s41598-023-39055-z)

**Differential gut microbiome in spondyloarthritis patients associated to *Blastocystis* colonization**

**Supplementary material**

**Supplementary table S1. Primers information**

Primers used to identify bacteria, *Entamoeba histolytica* and *Cryptosporidium*. Primers and probes used to identify *Blastocystis*, *Giardia intestinalis* and *Enterobacteriaceae*

| **Target gene** | **Organism, type of PCR, primer name** | | **Sequence 5´- 3´** | **Reference** |
| --- | --- | --- | --- | --- |
| ***18S rRNA*** | ***E. histolytica*** | |  |  |
|  | First PCR | E.histo-18S-F  EhR | GTTTGTATTAGTACAAAATGGCCAATTC  GATCTAGAAACAATGCTTCTCT | [1, 2] |
|  | Nested PCR | EntaF  EhR | ATGCACGAGAGCGAAAGCAT  GATCTAGAAACAATGCTTCTCT | [2] |
|  | ***Cryptosporidium*** | |  |  |
|  | First PCR | Crip_F  Crip_R | GGAAGGGTTGTATTTATTAGATAAAG  AAGGAGTAAGGAACAACCTCCA | [3] |
|  | Nested PCR | Chv_F  Crip_R | CAATAGCGTATATTAAAGTTGTTGCAGTT  AAGGAGTAAGGAACAACCTCCA | [4]  [3] |
|  | ***Blastocystis*** | |  |  |
|  | qPCR | Blasto_18S_F  Blasto_18S_R  Blasto_18S (probe) | GGTCCGGTGAACACTTTGGATTT  CCTACGGAAACCTTGTTACGACTTCA  FAM-TCGTGTAAATCTTACCATTTAGAGGA-BHQ1 | [5] |
|  | ***G. intestinalis*** | |  |  |
|  | qPCR | Giard_16S_F  Giard_16S_R  Giard_16S (probe) | CATGCATGCCCGCTCA  AGCGGTGTCCGGCTAGC  FAM-AGGACAACGGTTGCAC-BHQ1 | [1] |
| ***16S rRNA*** | ***Enterobacteriaceae*** | |  |  |
|  | qPCR | TTB_Forward  TTB_Reverse  TTB38K (probe) | AGAGTTTGATCMTGGCTCAG  TTACCGCGGCKGCTGGCAC  FAM-CCAKACTCCTACGGGAGGCAGCAG-BHQ1 | [6] |
|  | **All bacteria (region V3-V4)** | |  |  |
|  | PCR | Bakt_341F  Bakt_805R | CCTACGGGNGGCWGCAG  GACTACHVGGGTATCTAATCC | [7] |

**References**

1. Mejia, R., Vicuña, Y., Broncano, N., Sandoval, C., Vaca, M., Chico, M., Cooper, P. J., & Nutman, T. B. (2013). A novel, multi-parallel, real-time polymerase chain reaction approach for eight gastrointestinal parasites provides improved diagnostic capabilities to resource-limited at-risk populations. The American journal of tropical medicine and hygiene, 88(6), 1041–1047. https://doi.org/10.4269/ajtmh.12-0726
2. Hamzah, Z., Petmitr, S., Mungthin, M., Leelayoova, S., & Chavalitshewinkoon-Petmitr, P. (2006). Differential detection of Entamoeba histolytica, Entamoeba dispar, and Entamoeba moshkovskii by a single-round PCR assay. Journal of clinical microbiology, 44(9), 3196–3200. https://doi.org/10.1128/JCM.00778-06
3. Xiao, L., Morgan, U. M., Limor, J., Escalante, A., Arrowood, M., Shulaw, W., Thompson, R. C., Fayer, R., & Lal, A. A. (1999). Genetic diversity within Cryptosporidium parvum and related Cryptosporidium species. Applied and environmental microbiology, 65(8), 3386–3391. https://doi.org/10.1128/AEM.65.8.3386-3391.1999
4. Burnet, J. B., Ogorzaly, L., Tissier, A., Penny, C., & Cauchie, H. M. (2013). Novel quantitative TaqMan real-time PCR assays for detection of Cryptosporidium at the genus level and genotyping of major human and cattle-infecting species. Journal of applied microbiology, 114(4), 1211–1222. https://doi.org/10.1111/jam.12103
5. Stensvold, C. R., Ahmed, U. N., Andersen, L. O., & Nielsen, H. V. (2012). Development and evaluation of a genus-specific, probe-based, internal-process-controlled real-time PCR assay for sensitive and specific detection of Blastocystis spp. Journal of clinical microbiology, 50(6), 1847–1851. https://doi.org/10.1128/JCM.00007-12
6. Menu, E., Mary, C., Toga, I., Raoult, D., Ranque, S., & Bittar, F. (2018). Evaluation of two DNA extraction methods for the PCR-based detection of eukaryotic enteric pathogens in fecal samples. BMC research notes, 11(1), 206. https://doi.org/10.1186/s13104-018-3300-2
7. Herlemann, D. P., Labrenz, M., Jürgens, K., Bertilsson, S., Waniek, J. J., & Andersson, A. F. (2011). Transitions in bacterial communities along the 2000 km salinity gradient of the Baltic Sea. The ISME journal, 5(10), 1571–1579. https://doi.org/10.1038/ismej.2011.41

**Supplementary table S2. Intestinal parasites found in SpA patients and control individuals**

| **Intestinal Parasites** | **Diagnostic method** | **Total**  (n=49) | **SpA Patients**  (n=36) | **Controls**  (n=13) | ***p*-value*** | |
| --- | --- | --- | --- | --- | --- | --- |
|  |  | n | n - % | n - % |  |  |
| *Endolimax nana* | Microscopy | 41 | 29 - 80.56 | 12 - 92.31 | 0.353 | |
| *Blastocystis* | Microscopy and qPCR | 31 | 23 - 63.89 | 8 - 61.54 | 0.880 | |
| *Entamoeba coli* | Microscopy | 4 | 3 - 8.33 | 1 - 7.69 | 0.942 | |
| *Chilomastix mesnili* | Microscopy | 2 | 1 - 2.78 | 1 - 7.69 | 0.443 | |
| *Entamoeba hartmanni* | Microscopy | 1 | 1 - 2.78 | 0 | 0.544 | |
| *Giardia intestinalis* | Microscopy and qPCR | 0 | 0 | 0 | - | |
| *Entamoeba histolytica* | Microscopy and PCR | 0 | 0 | 0 | - | |
| *Cryptosporidium* | PCR | 0 | 0 | 0 | - | |
| Helminths | Microscopy | 0 | 0 | 0 | - | |
| (*****) Z-test proportions was used to calculate differences of proportions | | | | | |  |

**Supplementary table S3. Mean values of intestinal bacterial richness and diversity indices**

Means and standard deviation. ***(C):*** All control subjects. ***(P):*** All spondyloarthritis patients (SpA patients). ***(C -):*** Control subjects without *Blastocystis* ***(C +):*** Control subjects with *Blastocystis* ***(P -):*** SpA patients without *Blastocystis* ***(P +):*** SpA patients with *Blastocystis* ***Low:*** less disability according BASDAI or BASFI scores (values < 4). ***High:*** more disability according BASDAI or BASFI scores (values ≥ 4).

|  | **Mean values of intestinal bacterial richness and diversity indices** | | | | | | | | | |
| --- | --- | --- | --- | --- | --- | --- | --- | --- | --- | --- |
|  | **Control Subjects** | | | **SpA patients** | | | **BASDAI** | | **BASFI** | |
| **Index** | ***C*** | ***C-*** | ***C+*** | ***P*** | ***P-*** | ***P+*** | **Low** | **High** | **Low** | **High** |
| **Chao1** | 338 ± 91 | 266 ± 73 | 384 ± 70 | 270 ± 61 | 270 ± 70 | 270 ± 57 | 273 ± 77 | 270 ± 58 | 282 ± 63 | 265 ± 62 |
| **Shannon** | 7.7 ± 0.5 | 7.4 ± 0.4 | 7.9 ± 0.5 | 7.3 ± 0.3 | 7.3 ± 0.4 | 7.3 ± 0.3 | 7.2 ± 0.3 | 7.3 ± 0.4 | 7.3 ± 03 | 7.3 ± 0.4 |
| **Faith** | 19.2 ± 4.5 | 15.6 ± 3.2 | 21.4 ± 3.9 | 18.9 ± 4.6 | 19.2 ± 6.2 | 18.8 ± 3.6 | 20.6 ± 4.2 | 18.6 ± 4.7 | 20.4 ± 5.1 | 18.4 ± 4.3 |

**Supplementary table S4. Comparisons of mean relative abundance of microbial classes significantly different between individuals**

Taxa relative abundances were compared between subgroups and *p*-values significantly different are shown in bold. ***C-:*** Controls without *Blastocystis* ***C+:*** Controls with *Blastocystis* ***P-:*** Patients without *Blastocystis* ***P+***: Patients with *Blastocystis.* Statistical significance was obtained by White’s non-parametric t-test and Benjamini−Hochberg FDR correction (only taxa with *p*-value <0.05 and a DBM>0.3% are shown).

|  |  | ***p*-value** | | | |
| --- | --- | --- | --- | --- | --- |
| **Hierarchy** | **Taxa** | ***C-* vs *C+*** | ***P-* vs *P+*** | ***C-* vs *P-*** | ***C+* vs *P+*** |
| 1 | c_*Bacilli* | 0.923 | **0.030** | **0.033** | 0.607 |
|  | o_*Lactobacillales* | 0.721 | **0.030** | **0.013** | 0.582 |
|  | f_*Lactobacillaceae* | 0.255 | **0.040** | 0.076 | 0.159 |
|  | g_*Lactobacillus* | 0.227 | **0.040** | 0.063 | 0.234 |
|  | s_*Lactobacillus* *ruminis* | 0.324 | **0.040** | 0.084 | 0.368 |
| 2 | p_*Pseudomonadota* | 0.131 | 0.403 | 0.726 | **0.030** |
|  | c_*Gammaproteobacteria* | 0.676 | 0.383 | 0.935 | **0.010** |
|  | f_*Succinivibrionaceae* | 0.981 | 0.562 | 0.556 | **0.010** |
|  | g_*Succinivibrio* | 0.545 | 1.075 | 0.596 | **0.010** |
| 3 | f_*Clostridiaceae* | 0.773 | **0.020** | 0.173 | 0.259 |
|  | g_*Clostridium* | 0.790 | **0.010** | 0.168 | 0.259 |
| 4 | g_*Catenibacterium* | **0.050** | 0.164 | **0.019** | 0.453 |
|  | s_*Catenibacterium* *mitsuokai* | **0.041** | 0.174 | **0.013** | 0.473 |
| 5 | f_*Veillonellaceae* | **0.050** | 0.652 | 0.075 | 0.751 |

**Supplementary figure S1. Taxonomic characterization for gut microbiome in SpA patients and control individuals, colonized and not colonized by *Blastocystis***

Panels **a, b** and **c** show the average relative frequency at order, class and phylum level, respectively. Taxa with relative frequency greater than 0.5% are shown. ***C-***: Control subjects *Blastocystis* free (n=5). ***C+***: Control subjects colonized by *Blastocystis* (n=8). ***P-***: SpA patients *Blastocystis* free. (n=13). ***P+***: SpA patients colonized by *Blastocystis* (n=23).


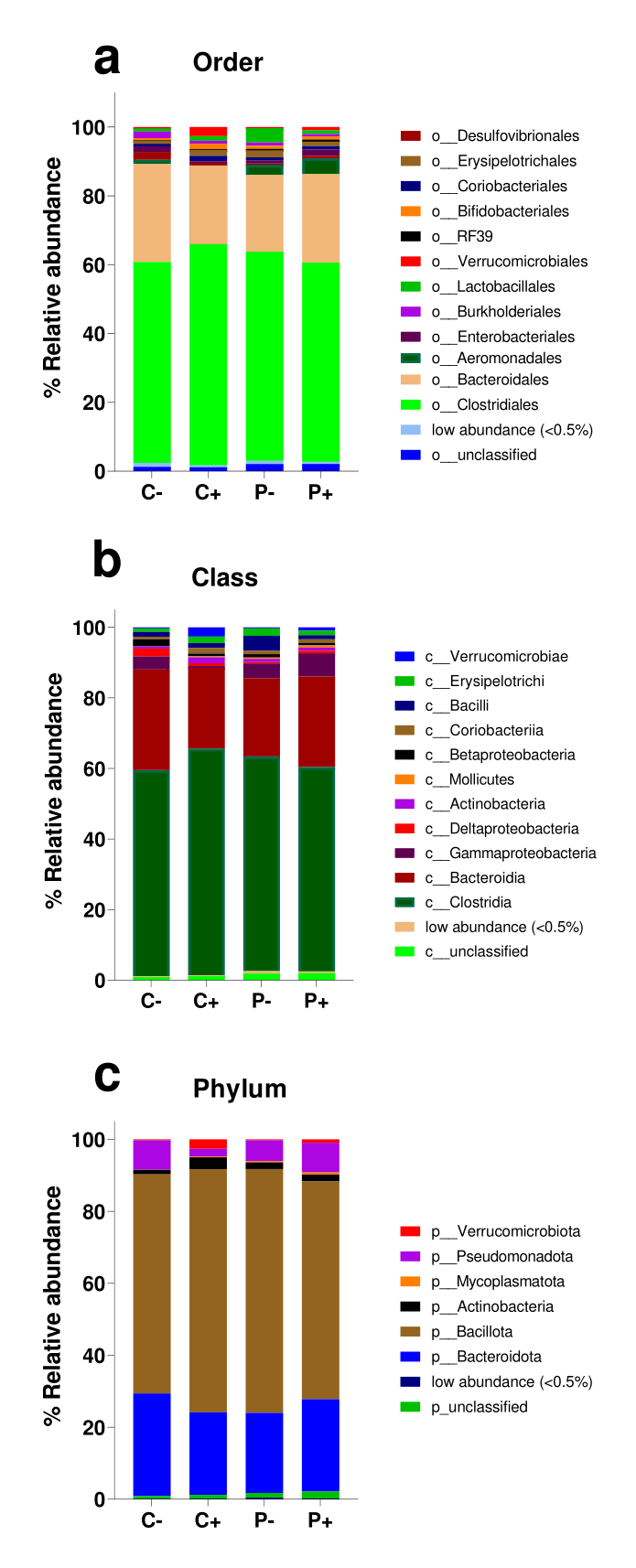


**Supplementary figure S2. Beta diversity of gut microbiome in control individuals and SpA patients according to BASDAI and BASFI indices.**

**(a)** and **(c)**: Bray-Curtis dissimilarity Principal Coordinate Analysis (PCoA) based on bacteria community features between control subjects and SpA patients according to BASDAI and BASFI indices, respectively. **(b)** and **(d)**: PERMANOVA analysis was used to compare differences in beta diversity between groups. Significant differences (*p*-value <0.05) are shown in bold. ***C:*** control individuals. ***Low*:** BASDAI or BASFI indices < 4 (less disease activity or functional limitation). ***High:*** BASDAI or BASFI indices ≥ 4 (more disease activity or functional limitation).


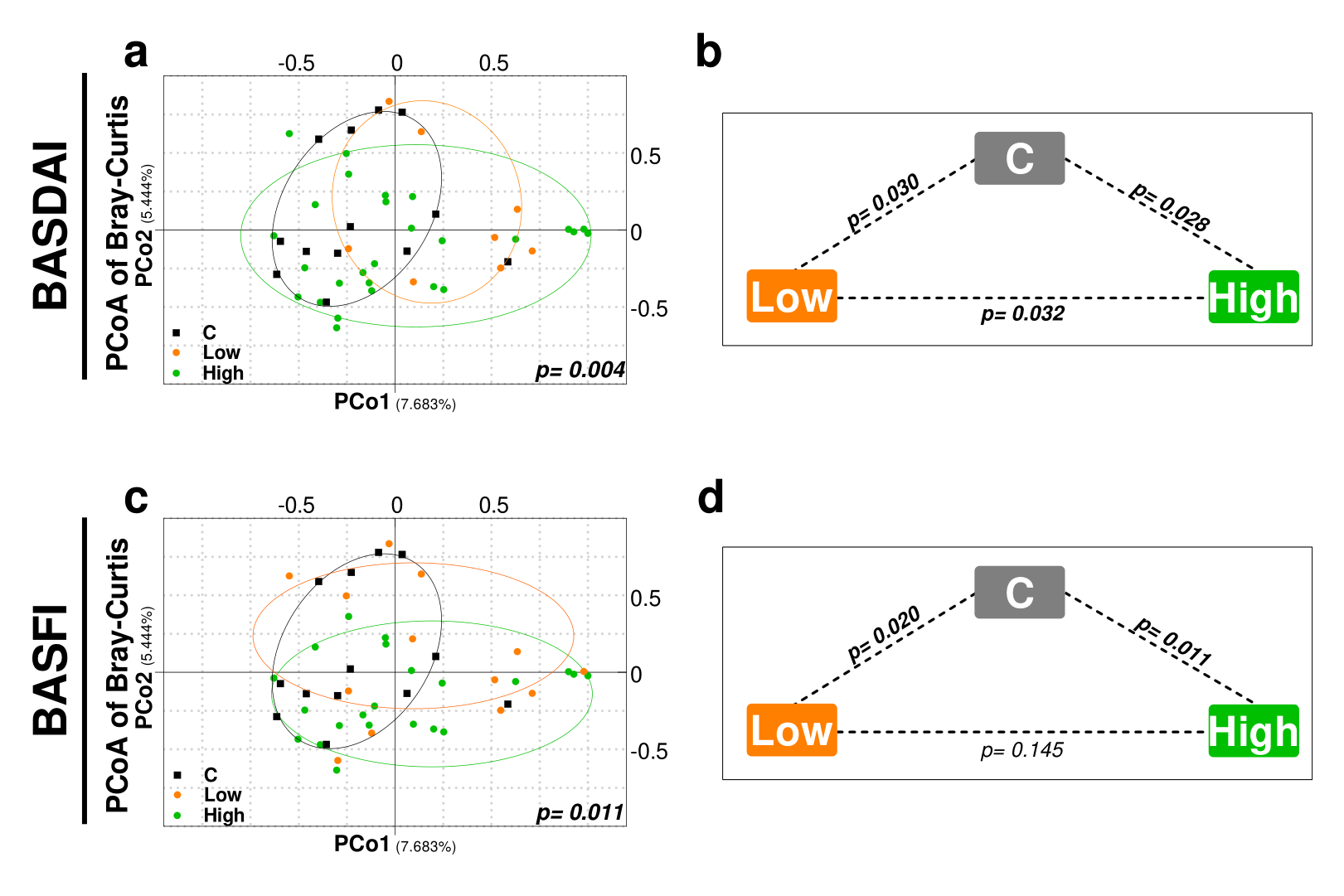

Supplement: Supplementary file 1 — Supplementary Information. [file 41598_2023_39055_MOESM1_ESM.docx]
